# Anxiety disorders and Quality of life: The Role of Occupational Therapy

**DOI:** 10.1192/j.eurpsy.2024.881

**Published:** 2024-08-27

**Authors:** V. Moraiti, A. Kalmanti, A. Papadopoulou, G. N. Porfyri

**Affiliations:** ^1^Early Intervention in Psychosis (EIP), Ikelos NGO (Collab. Attikon University Hospital); ^2^2nd Department of Psychiatry, Medical School, National and Kapodistrian University of Athens, “Attikon” University Hospital; ^3^2nd Department of Psychiatry, Medical School, National and Kapodistrian University of Athens, “Attikon” University Hospital, Athens; ^4^Psychiatric Department, General Hospital of Papanikolaou, Thessaloniki, Greece

## Abstract

**Introduction:**

Anxiety disorders represent the most common mental illnesses, which are listed among the ten most important causes of disability worldwide. According to DSM-5, they are defined as “disorders that share characteristics of excessive fear and anxiety and related behavioral disorders”. Patients exhibit low levels of quality of life. Their daily routine is affected negatively. However, Occupational Therapy has been proven to play a crucial role in their treatment, improving quality of life through the involvement in occupations.

**Objectives:**

To highlight the contribution of Occupational Therapy in ameliorating the quality of life in anxiety disorders.

**Methods:**

A review of 50 articles -from 2013 to 2023- on PubMed and Google Scholar, regarding the beneficial impact of Occupational Therapy in the Anxiety Disorders’ treatment.

**Results:**

Occupational Therapists can intervene in many negatively affected -by the disease- life domains such as: Activities of Daily Living, Education, Work, Play, Social Interaction and Sleep. The most effective Occupational Therapy methods are based on the cognitive behavioral approach and include: Psychoeducation, Relaxation techniques, Social skills training and Systematic desensitization.

Other methods involve training in Activities of Daily Living such as feeding, maintaining good personal hygiene, and using public transport. Furthermore, Art Therapy (visual arts, use of clay) has been shown to reduce feelings of anxiety, while promoting creativity and enhancing self-esteem.

**Conclusions:**

Additional research is needed regarding the effectiveness of Occupational Therapy in improving the quality of life for patients suffering from Anxiety Disorders. The important “take home message” is that the amelioration of the patients’ quality of life should be the main goal of the therapeutic intervention and not a secondary result of it.

**Disclosure of Interest:**

None Declared

